# Effect of connector size and configuration on the fracture resistance of a long-span implant-supported monolithic zirconia fixed partial denture (An in vitro Study)

**DOI:** 10.1186/s12903-025-06743-3

**Published:** 2025-08-26

**Authors:** Alaa M. Khorshed, Lomaya Ghanem, Tamer E. Shokry

**Affiliations:** 1 Faculty of oral and Dental Medicine, Misr International University, Cairo, Egypt; 2 Conservative Dentistry Department, Faculty of oral and dental Medicine, Misr International university, Cairo, Egypt; 3 Fixed prosthodontics Department, Faculty of Dental Medicine, Al-Azhar University, Cairo, Egypt

**Keywords:** Monolithic zirconia, Fixed partial denture, Fracture resistance, Connector size, Connector design

## Abstract

**Background:**

Monolithic zirconia fixed partial dentures (FPDs) have gained popularity due to their high strength, biocompatibility and reduced veneer chipping compared to bilayered restorations. However, the connector region remains a critical point of mechanical failure, especially in long span implant supported FPDs subjected to high masticatory forces. The purpose of this in vitro study was to compare the fracture resistance of implant-supported Y-TZP (yttria-stabilized tetragonal zirconia polycrystal) fixed partial dentures (FPDs) with two connector sizes (9 mm² and 12 mm²) and two connector cross-sectional shapes (oval and circular).

**Methods:**

Twenty-eight four-unit posterior implant-supported monolithic zirconia FPDs were fabricated. They were divided into two equal groups (*n* = 14) based on connector cross-sectional shape (oval vs. circular), each further subdivided by connector size (9 mm² or 12 mm², *n* = 7 per subgroup). A static fracture test was performed by applying an occlusal load at a crosshead speed of 1 mm/min until failure, and the fracture resistance (load to fracture) was recorded. The data distribution was first checked with the Kolmogorov-Smirnov and Shapiro-Wilk tests, which confirmed that the fracture resistance values followed a normal (parametric) distribution. A two-way analysis of variance (ANOVA) was performed to assess the effects of the connector size (9 mm² vs. 12 mm²), connector cross-section (oval vs. circular), and their interaction on the mean fracture resistance. The significance level was set at α = 0.05.

**Results:**

Regardless of cross-sectional shape, the 9 mm² connector groups showed significantly lower mean fracture resistance than the 12 mm² connector groups. Regardless of connector size, the oval cross-section groups showed significantly higher mean fracture resistance than the circular groups.

**Conclusion:**

Based on the findings of this in vitro study, it can be concluded that increasing the connector cross-sectional area and using an oval connector shape both enhance the fracture resistance of long-span monolithic zirconia FPDs.

## Clinical implications

Evaluating the influence of connector size and cross-sectional shape on the fracture resistance of four-unit implant-supported monolithic zirconia FPDs can guide clinicians in selecting connector dimensions that optimize durability and esthetics. In particular, using a larger connector size (12 mm²) and an oval connector shape may enhance the long-term success of zirconia fixed prostheses by improving load distribution and reducing the risk of fracture.

## Introduction

The clinical use of implant-supported all-ceramic fixed partial dentures (FPDs) to restore missing teeth has increased dramatically due to their predictable performance in mechanical strength, esthetics, and biocompatibility. Implants can be used to replace a single tooth or span short or long edentulous areas of up to two missing teeth [1, 2]. 

Ceramic materials have gained popularity, particularly for restorations in the esthetic zone. Several types of dental ceramics with favorable esthetic and biocompatible properties have been introduced. Traditional bilayered zirconia restorations offer excellent mechanical properties; however, failures such as cohesive fracture or delamination at the veneer–core interface have been reported [3–5]. 

The need to overcome the problem of veneer chipping in Y-TZP restorations led to the development of full-contour (monolithic) zirconia. Since the introduction of monolithic zirconia for partial dentures, the overall failure rate has been significantly lower over five-year observation periods [6–8]. 

Monolithic Y-TZP restorations are typically fabricated by computer-aided design and computer-aided manufacturing (CAD/CAM) technology. FPDs made with CAD/CAM have been shown to withstand higher loads than those made by conventional techniques [9, 10]. 

All-ceramic FPDs typically fracture in the connector region, initiating at the gingival embrasure and moving all the way to the occlusal surface. This phenomenon can be explained by the fact that FPD supported by an all-ceramic implant behaves like a ceramic beam on rigid supports when subjected to masticatory stresses. The prosthesis’s occlusal surface experiences compressive stresses during mastication. Tensile stress is thus concentrated in the connections’ area by the narrow constrictions with asymmetric irregular forms. As a brittle material, ceramics tend to fail in tension by a lack of ductility [11]. 

For optimal esthetic, biologic, and functional outcomes, the connector area of a splinted FPD should be as small as possible to create natural-appearing embrasures. However, the connector must also be large enough to ensure adequate mechanical strength for zirconia. The ideal connector design allows the units of the FPD to appear separated (improving esthetics) while still providing sufficient bulk for strength. A very small connector cross-section can achieve excellent esthetics but may compromise mechanical properties [12]. 

The appropriate connector size and shape for long-span zirconia FPDs remains a subject of debate. Larsson et al. tested four-unit Y-TZP FPDs with various connector sizes (2.0, 2.5, 3.0, 3.5, and 4.0 mm) and recommended a minimum connector diameter of 4 mm. Finite element analyses have similarly indicated that increasing connector height (and thus cross-sectional area) reduces the stresses experienced by the connector [13–15]. 

Achieving both high esthetic appeal and acceptable mechanical performance is therefore a challenge for the clinician, especially in stress-bearing areas like the molar region. For an implant-supported FPD with a limited occluso-gingival height such as the anterior region, reducing the connector size to improve esthetics can markedly increase stresses and risk of failure [16–20]. 

The purpose of this study was to compare the fracture resistance of implant-supported Y-TZP four-unit FPDs with two different connector sizes (9 mm² and 12 mm²) and two connector cross-sectional configurations (oval and circular). The null hypothesis was that connector size and shape would have no influence on the fracture resistance of a monolithic zirconia FPD spanning two missing mandibular posterior teeth.

## Methods

Based on the results of Hafezeqoran et al. [19], an effect size f ≈ 0.68 was estimated for fracture resistance. Using an α = 0.05 and β = 0.20 (power = 80%), the required total sample size was calculated to be 28 specimens (7 per subgroup). The sample size calculation was performed using G*Power (v3.1.9.7) [21]. 

This in vitro study evaluated the fracture resistance of a total of 28 four-unit posterior implant-supported FPDs fabricated from monolithic zirconia. The FPDs were divided into two equal groups (*n* = 14) based on connector cross-sectional shape (oval vs. circular). Each shape group was further subdivided into two connector size groups: 9 mm² cross-sectional area and 12 mm² cross-sectional area (*n* = 7 specimens per subgroup). (Table 1).

**Table 1:** Factorial design

Connector configuration			Total
Shape	Dimensions		
Oval	(3 × 4)12mm^2^ (*n* = 7)	(3 × 3) 9mm^2^ (*n* = 7)	14
Circular	**(3 × 4)12 mm**^**2**^ (***n*** = 7)	**(3 × 3) 9 mm** ^**2**^ (***n*** = 7)	**14**
Total	**14**	**14**	**28**

**Fig. 1:**
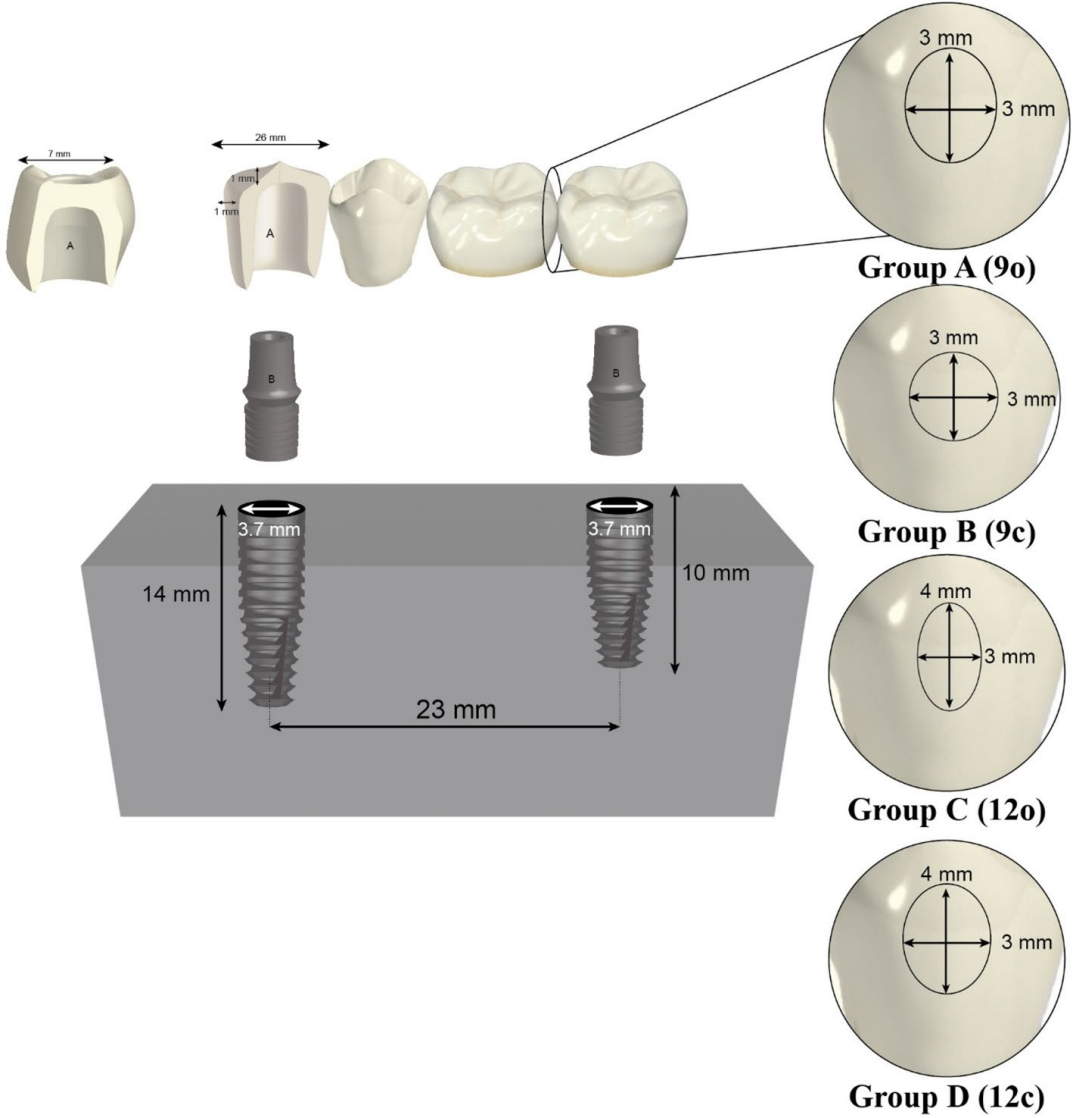
Diagram representing the master model with the 4-unit monolithic zirconia framework and 4 different connector designs

One stainless steel master model was used for each group and it was custom-designed to simulate a section of the mandible with the second premolar and first molar missing. Two holes were drilled in the model to accommodate the implants. Two implants (Dual Implant system, Titan industries, Egypt) were placed in the model to represent the missing premolar and molar positions. (Table 2) shows the materials used in this study. A dental surveyor (Bredent BF-2; Weissenhorn, Germany) was used during implant placement to ensure the implants were parallel and oriented vertically in the model [22]. The center-to-center distance between the two implants was ~ 23 mm, corresponding to the average distance between a lower first premolar and second molar [23]. Schematic Diagram is presented in (Fig. 1).

**Table 2:** Materials used in the study

Serial	Brand Name	Description	Manufacturer	Composition	Lot Number
1	Dual Implant	Implants: Diameter 3.7 mm, Length 10 mm Diameter 3.7 mm, Length 14 mm	Titan Industries EG (Egypt)	Grade 23 titanium (Ti6AL-4VE.L.I)	B0050724
2	Dual Implant	Abutments: straight, contoured, ready-made abutments	Titan Industries EG (Egypt)	Grade 23 titanium (Ti6AL-4VE.L.I)	D0360224
3	Katana	Strength gradient monolithic zirconia disc	Kurary Noritake (Japan)	ZrO2 88.0%−95.5%, Y2O3 > 4.5%- ≤7.0%, AL2O3 ≤ 1.0%, HfO2 ≤ 5.0%, other oxides ≤ 1.5%	EISPQ
4	Es Temp	Spident EsTemp Implant Temporary Resin Cement	Spident (korea)	-Base Zinc Oxide -Catalayst-Rosin, Nonanoic Acid	ET22080
5	BILKIM 3D Scanning Spray	3D Scanning Spray	BILKIM (Turkey)	TiO_2_ + C_5_H_12_ + C_6_H_12_+SiO_2_	2225

Straight, ready-made titanium abutments (Dual Implant system) were attached to each implant and tightened to 35 N using a calibrated torque wrench. The implant–abutment assemblies were fixed in the model by positioning them on the surveyor and pouring self-curing methacrylate-based resin (auto-polymerizing resin) around them. The resin (mixed per manufacturer’s instructions) was introduced into the drilled holes and the implants with abutments were lowered into position [24]. The resin was allowed to polymerize completely, securing the implants rigidly in the stainless-steel block. Excess resin was removed flush with the surface.

**Fig. 2:**
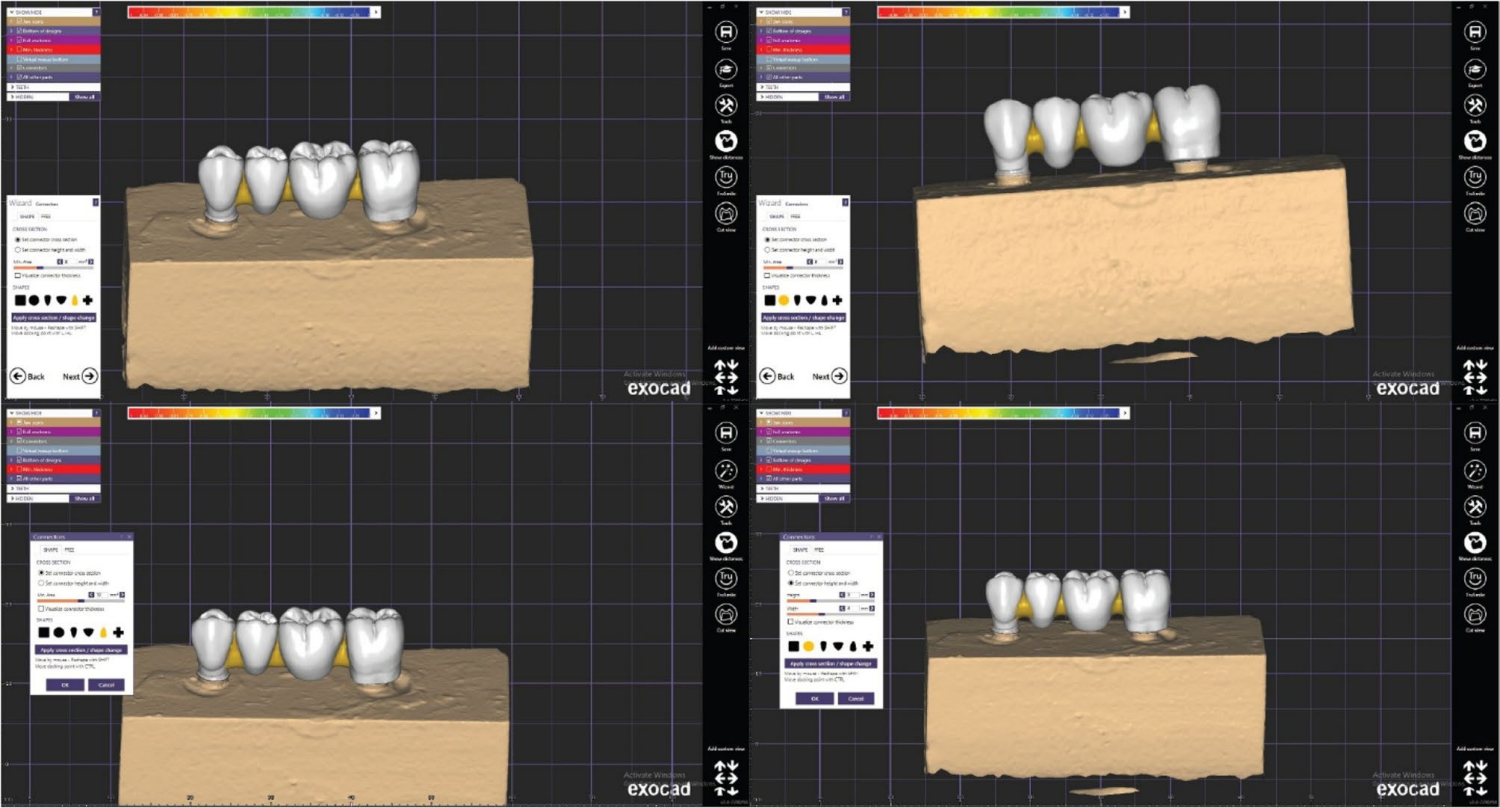
Frameworks on Exocad program for designing process

Before digitizing the model, a thin coat of 3D scanning spray (BILKIM, Turkey) was applied to eliminate surface glare. The master model was then scanned using a laboratory scanner (Medit i700, Seoul, South Korea) [25]. The four-unit FPD frameworks were designed using CAD software (exocad DentalCAD v2.3, exocad GmbH). (Fig. 2). The framework was designed with an occlusal thickness of 1 mm and an axial thickness of 1 mm, and a uniform cement spacer of 100 μm [26]. The pontics (spanning the first molar and second premolar spaces) were designed to be approximately 10 mm occluso gingival height, 7 mm in bucco-lingual width and to fill the ~ 20 mm mesio-distal edentulous span. Four distinct connector designs were created corresponding to the four test groups (connector shape × size).

**Fig. 3:**
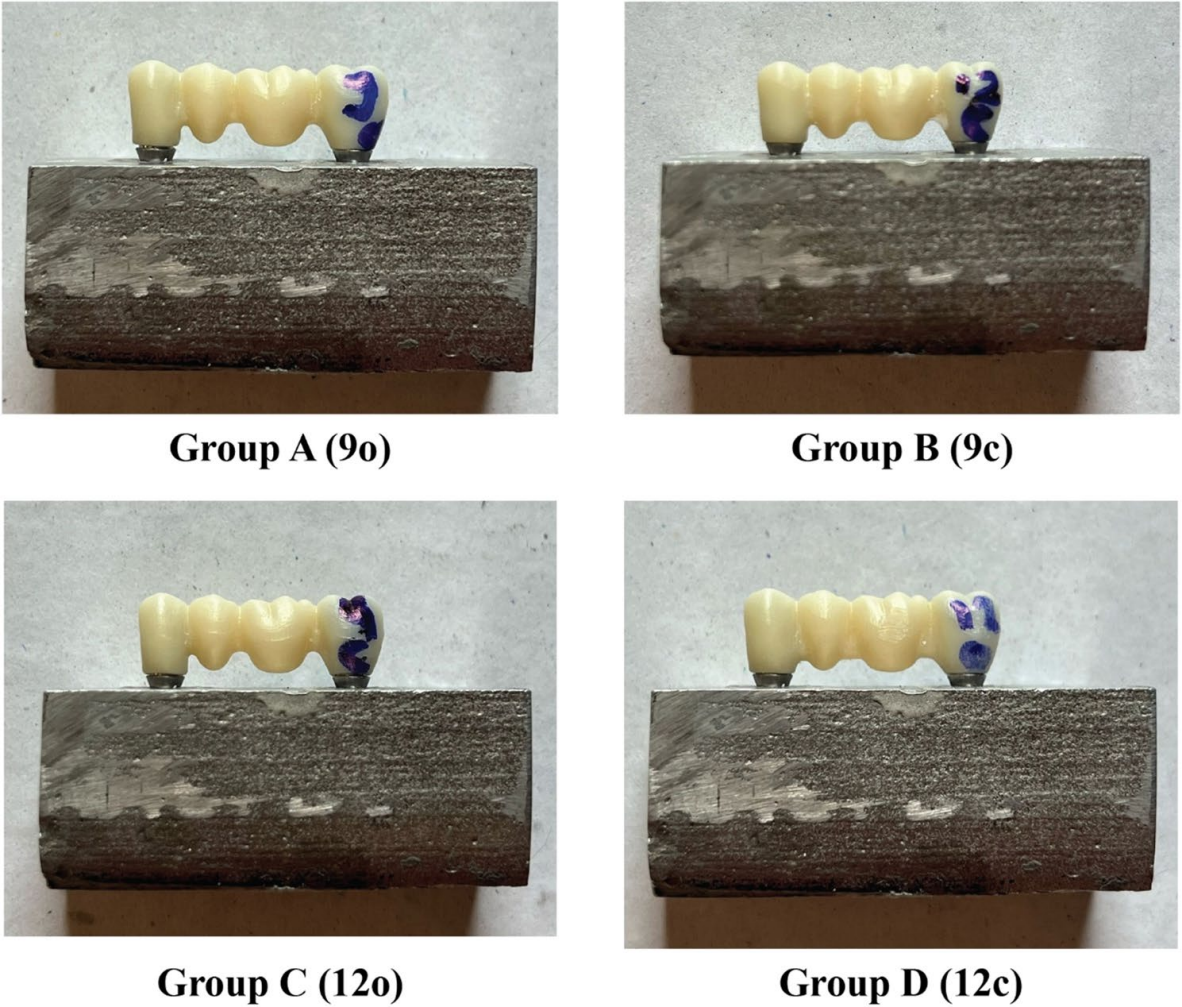
Frameworks on master model representing group A −9o (3 × 3mm^2^/oval cross section) group B-9c (3 × 3mm^2^/circular cross section) group C-12o (3 × 4mm^2^/oval cross section) group D- 12c (3 × 4mm^2^/circular cross section)

After the frameworks were designed and approved in the CAD software, the STL design files were exported to the CAM system. All 28 FPD frameworks were milled from a Katana zirconia disc using a 5-axis milling machine (DWX-51D; Roland DG Corp., Japan). The milled frameworks were then sintered to full density at 1500 °C for 7 h in a programmable sintering furnace (inLab Profire; Dentsply Sirona) following the zirconia manufacturer’s recommended sintering cycle. Frameworks after milling are presented in (Fig. 3).

**Fig. 4:**
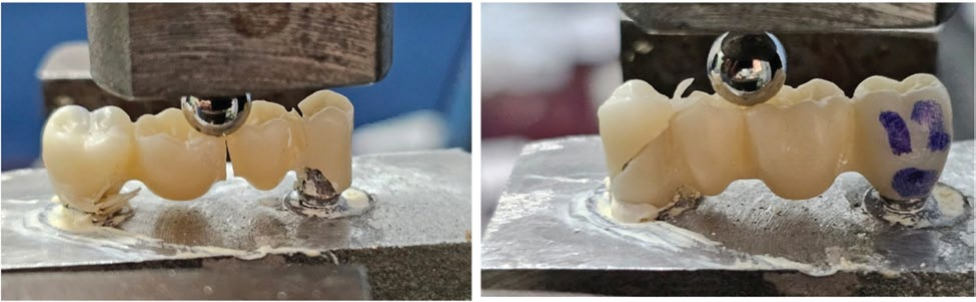
Frameworks after fracture test subjected to static loading using universal testing machine

Each zirconia FPD was luted onto its implants using a temporary resin cement (Spident EsTemp Implant) to allow for easy removal and reuse of the model between tests. All samples were then individually mounted in a universal testing machine (Instron Model 3345, Norwood, MA, USA) with a 5 kN load cell. A compressive load was applied occlusally to the center of pontic area of each FPD using a metallic rod with a spherical tip 5 mm in diameter. The load was applied at a crosshead speed of 1 mm/min until catastrophic failure of the FPD. Frameworks after fracture test are presented in (Fig. 4).

After fracture testing, the fracture surfaces of all specimens were examined by scanning electron microscopy (SEM) to perform a fractographic analysis. Prior to SEM (JCM-7000 NeoScope™ Benchtop SEM) observation, the fractured surfaces were cleaned in an ultrasonic bath with ethyl alcohol to remove debris, then rinsed with distilled water. (Acetone and methanol were avoided as they can leave residues on the surface.) Once cleaned, the fracture surfaces were dried and sputter-coated with a gold film (~ 10–15 nm thick) for 5 min at 15 mA to improve surface conductivity. Each specimen was then mounted on an SEM stub using carbon adhesive [27, 28]. 

The specimens were examined under SEM at magnifications of approximately 50×, 100×, 200×, and 400×. Working distance used was 10 mm as it gives higher resolution and better surface detail. Accelerating voltage used was 15 kv being a commonly selected standard for balance between resolution and compositional analysis. This allowed identification of fractographic features such as the origin of the crack, the direction of crack propagation, and the presence of hackle lines or other indicative markings on the fracture surface.

### Statistical analysis

Fracture resistance data (maximum load to fracture) were analyzed statistically. The data distribution was first checked with the Kolmogorov-Smirnov and Shapiro-Wilk tests, which confirmed that the fracture resistance values followed a normal (parametric) distribution. Therefore, parametric tests were used. Mean and standard deviation (SD) were calculated for each group. A two-way analysis of variance (ANOVA) was performed to assess the effects of the connector size (9 mm² vs. 12 mm²), connector cross-section (oval vs. circular), and their interaction on the mean fracture resistance. When the ANOVA indicated a significant effect, pairwise comparisons were conducted using Bonferroni post hoc tests. The significance level was set at α = 0.05. All statistical analyses were performed using IBM SPSS Statistics for Windows (v23.0; IBM Corp., Armonk, NY, USA).

## Results

Two-way ANOVA revealed that connector size and connector cross-section each had a statistically significant effect on the mean fracture resistance of the FPDs (*P* ≤ 0.01), while the interaction between size and cross-section was not statistically significant (*P* > 0.05). In other words, larger connectors and oval-shaped connectors both tended to increase fracture resistance, and their effects were additive (no significant interaction). The two-way ANOVA results are summarized in (Table 3).

**Table 3:** Two-way ANOVA results for the effect of different variables on mean fracture resistance (Newton)

Source of variation	Type III Sum of Squares	df	Mean Square	F-value	*P*-value	Effect size (Partial eta squared)
Connector size	394468.6	1	394468.6	9.1	0.006*	0.275
Cross-section	595173.1	1	595173.1	13.8	0.001*	0.364
Connector size x cross-section interaction	181208.8	1	181208.8	4.2	0.052	0.149

*df *degrees of freedom* = (n-1)*

*Significant at *P* ≤ 0.05

Regardless of cross-sectional shape, the FPDs with 9 mm² connectors showed a significantly lower mean fracture resistance than those with 12 mm² connectors. Similarly, regardless of connector size, FPDs with an oval connector cross-section had a significantly higher mean fracture resistance than those with a circular cross-section. These findings indicate that larger connector size and oval connector shape each improve the fracture resistance. Because the interaction term was not significant, the effects of size and shape can be considered independently.

**Fig. 5:**
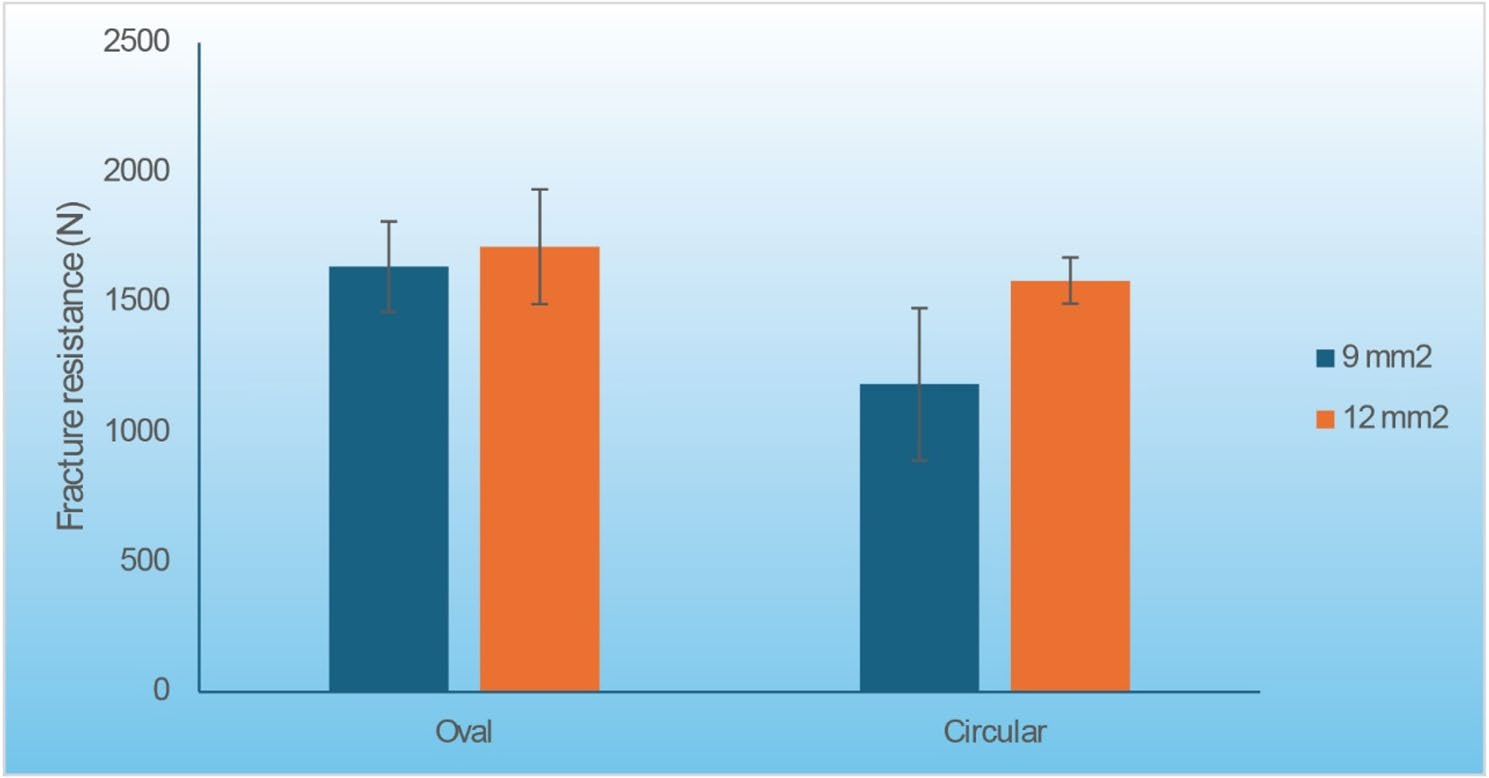
Bar chart representing mean and standard deviation values for fracture resistance with different interactions of variables

Although the interaction was not statistically significant, a post hoc comparison of subgroups was conducted to illustrate the trend. With an oval connector cross-section, there was no significant difference in fracture resistance between the 9 mm² and 12 mm² connectors. In contrast, with a circular cross-section, the 9 mm² connectors had significantly lower fracture resistance than the 12 mm² connectors. Likewise, for the 9 mm² connector groups, the oval cross-section yielded significantly higher fracture resistance than the circular cross-section, whereas for the 12 mm² connector groups, there was no significant difference between oval and circular cross-sections. The mean fracture resistance values for each group and the results of pairwise comparisons are presented in (Table 4). Bar chart presenting mean and standard deviation values are presented in (Fig. 5).

**Table 4:** The mean, standard deviation (SD) values and results of two-way ANOVA test for comparison between fracture resistance (Newton) with different interactions of variables

Cross-section	9 mm^2^		12 mm^2^		*P*-value	Effect size (Partial eta squared)
	Mean	SD	Mean	SD		
Oval	1636.6	174.8	1713.1	221	0.498	0.019
Circular	1184.1	292.6	1582.4	89.3	0.002*	0.348
*P*-value	<0.001*		0.251			
Effect size *(Partial eta squared)*	0.408		0.054			

*Significant at *P* ≤ 0.05

### Fractographic analysis

**Fig. 6:**
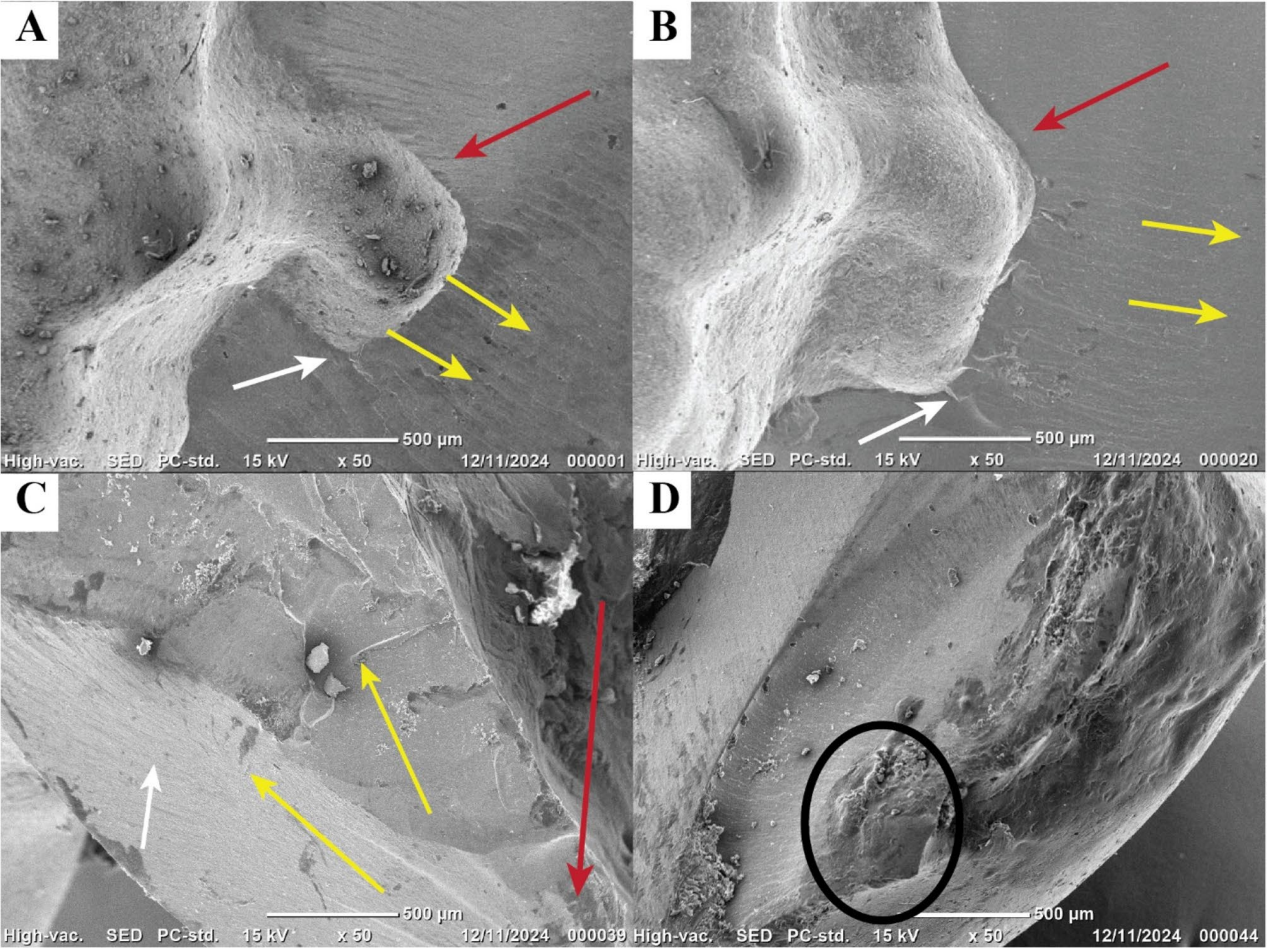
Scanning electron microscope images of fracture surfaces subjected to static loading at magnification x50. Image A presenting group 9c (3 × 3mm^2^/circular cross section) Image B presenting group 9o (3 × 3mm^2^/oval cross section) Image C presenting group 12c (3 × 4mm^2^/circular cross section) Image D presenting group 12o (3 × 4mm^2^/oval cross section). A red arrow in the SEM image indicates the crack origin on the gingival side of the connector. A yellow arrow shows the direction of crack propagation toward the occlusal (coronal) part, and white arrows denote wake hackle lines following the crack path

Fractographic analysis under SEM was used to examine the fracture origin and crack propagation in each specimen. Both sides of each fractured FPD were inspected for consistent features. Key fractographic features (crack origin, crack propagation direction, hackle lines, etc.) were identified to better understand the failure patterns (Fig. 6).

- Fig. 6 (A) (Group 9 C): Fracture surface from a 9 mm² circular-connector sample. The fracture originated at the connector region. A red arrow in the SEM image indicates the crack origin on the gingival side of the connector. A yellow arrow shows the direction of crack propagation toward the occlusal (coronal) part, and white arrows denote wake hackle lines following the crack path.
- Fig. 6 (B) (Group 9O): Fracture surface from a 9 mm² oval-connector sample. The fracture features are similar to Figure A, with the crack origin in the connector region (red arrow) and propagation upward (yellow arrow). In this sample, multiple pronounced wake hackle lines (white arrows) are visible, indicating the crack’s advance through the connector.
- Fig. 6 (C) (Group 12 C): Fracture surface from a 12 mm² circular-connector sample. In this case, the crack initiated in the retainer (crown) area rather than the connector. The red arrow marks the crack origin in the crown, the yellow arrow shows the crack propagation direction, and the white arrows indicate wake hackle lines.
- Fig. 6 (D) (Group 12O): Fracture surface from a 12 mm² oval-connector sample. The crack originated in the retainer area as well. The fracture surface shows a transition from a smooth area to a rough, torn area, characteristic of a compression curl. The blue circle highlights this compression curl – a curved lip on the compression side of the fracture, indicating that bending forces were involved before final failure. (Red and yellow arrows are not present in this image, as the origin is marked by the blue circle).

Notably, in the 9 mm² connector groups (Fig. 6A and B), the fracture origin was at the connector, whereas in the 12 mm² groups (Fig. 6C and D), the fracture originated in the retainer portion of the FPD. In other words, the weaker point of the 9 mm² connector FPDs was the connector itself, while the 12 mm² connector made the retainer (crown) the next most vulnerable region. Figure 6A and B correspond to failures at the connector region, while Fig. 6C and D show failures at the retainer region.

## Discussion

Increasing esthetic demands have prompted clinicians to use all-ceramic prostheses to replace missing teeth. In this regard, zirconia is one of the strongest esthetic restorative materials available [29]. The present study assessed the effect of different connector sizes and shapes on the fracture resistance of a long-span monolithic zirconia FPD. The null hypothesis—that connector configuration would have no effect—was rejected, as both a larger connector size and an oval connector shape significantly improved fracture resistance.

The choice of using a monolithic zirconia framework (without a porcelain veneer) proved advantageous for load capacity. No veneering porcelain was added in our specimens in order to eliminate the variable of veneer chipping and to standardize the test bodies; veneer chipping is a common failure mode in layered zirconia restorations under stress [30]. By using monolithic Y-TZP, we focused the investigation on the core material’s behavior without the complications of a veneering layer.

Methodologically, a stainless-steel master model was used instead of a resin or epoxy model to ensure sufficient strength and rigidity during fracture loading. One model for each group which is custom-made was utilized for all tests to maintain consistency across specimens. This approach is in accordance with Negm et al. [3] who employed a standardized stainless-steel model to test the fracture resistance of four-unit zirconia FPDs.

The digital workflow and fabrication techniques were also chosen for precision and consistency. The Medit i700 scanner was used to capture the model digitally; this choice is supported by Ali et al. [25]who found the Medit i700 to have high trueness compared to other intraoral scanners. CAD/CAM technology allowed us to accurately control the connector dimensions and shapes as per the experimental design. All FPDs were milled from a single batch of high-quality zirconia to avoid material inconsistencies.

During testing, a temporary luting cement was used to seat the restorations. This facilitated easy removal and replacement of the FPDs between tests and ensured that the failure occurred in the zirconia framework rather than at the implant–abutment interface. The implants were embedded in acrylic within the metal block for stability, and a surveyor was utilized to position them correctly, ensuring a consistent path of insertion and alignment for each FPD. The fracture load was applied in a controlled manner on the pontic, reflecting a worst-case scenario of load concentration on the middle of the span.

In this study, seven FPD specimens were tested for each condition (9O, 9 C, 12O, 12 C), providing a basis for statistical comparison. The general fracture patterns observed were instructive: in the 9 mm² connector groups, cracks consistently originated at the connector, whereas in the 12 mm² groups, the initial failure tended to occur at the retainers (the crowns on the terminal abutments). This suggests that the 9 mm² connector was the weakest link in those FPDs, but when the connector size was increased to 12 mm², the connector was no longer the limiting factor—instead, the stress shifted to the next weakest component (the crown portion). The fractographic analysis confirmed these observations, showing distinct origin locations for the two sizes (connector vs. retainer).

All failures were characterized as brittle fractures, which is expected for zirconia ceramic. Zirconia, like most ceramics, is strong under compression but prone to crack propagation under tensile stress, and it fails without plastic yielding. This brittle behavior was evidenced by the nature of the fracture surfaces and the absence of any plastic deformation [28]. 

All fractures exhibited a brittle fracture mode, as expected for ceramic materials. Brittle materials like zirconia undergo minimal plastic deformation (on the order of 0.01% strain) before fracturing, and they are strong in compression but weak in tension [28]. The presence of hackle lines on many fracture surfaces (visible as parallel lines radiating from the origin) further confirmed the brittle nature of the failure. These hackle lines trace the local direction of crack propagation. Wake hackle lines, which branch off from the main crack front after encountering a heterogeneity (such as a pore or grain boundary), were observed particularly in the 9O group (Fig. 6B); they reliably indicate the crack path and direction. The observation of a compression curl in the 12O group (Fig. 6D) suggests that a bending component was present: as the crack propagated, the compressive side of the bending beam formed a curled lip just before final separation. Together, these fractographic features allowed us to identify the crack origins and propagation directions for each failure, confirming that larger connectors shifted the weakest link away from the connector and into the retainers.

The fracture resistance findings demonstrated that both increased connector size (12 mm² vs. 9 mm²) and an oval cross-sectional shape significantly enhanced the mechanical performance of monolithic zirconia FPDs. These mechanical outcomes were strongly supported by the fractographic analysis. SEM images revealed that in the lower-strength 9 mm² groups, fractures consistently originated at the gingival side of the connector—confirming it as the weakest point. Conversely, in the 12 mm² groups, failure shifted away from the connector and initiated in the retainer crown, indicating that the larger connector effectively redistributed stress and reinforced the connector area. The presence of wake hackle lines in smaller connectors and compression curls in larger ones further underscored these patterns of stress concentration and crack propagation. Thus, the fractographic evidence aligns with the mechanical data, confirming that increasing the connector size and optimizing its shape not only raises fracture resistance but also alters the failure origin, enhancing the structural reliability of long-span zirconia FPDs.

Using a larger connector size (12 mm² vs. 9 mm²) had a clear beneficial effect on fracture resistance. A 12 mm² connector provides greater cross-sectional area and structural rigidity, effectively adding support to the pontic and distributing occlusal forces more evenly across the FPD. This is especially important in posterior restorations, which encounter higher masticatory forces. By contrast, a 9 mm² connector concentrates stress more intensely, which can lead to higher tensile stress within the connector and predispose the FPD to earlier failure.

The improvement observed with 12 mm² connectors aligns with fundamental engineering principles and has been supported by other research. For instance, studies have shown that increasing the connector’s cross-sectional area significantly enhances the load-bearing capacity of zirconia FDPs. This is because larger connectors distribute occlusal forces over a greater volume of material, thereby reducing stress per unit area [19]. Similarly, the oval connector shape has been found to outperform the circular shape in terms of fracture resistance. This can be attributed to the fact that an oval shape distributes stress more efficiently over a broader vertical plane, whereas a circular cross-section tends to concentrate stress centrally. These findings are consistent with prior research suggesting that increasing the height of the connector or adopting an oval shape improves the longevity of FPDs [12]. Another study compared 3-unit, 4-unit, and 5-unit zirconia FDPs and recommended minimum connector cross-sectional areas to maintain a failure probability below 5% over a 20-year period. Specifically, it was suggested that 3-unit FDPs require at least 5.7 mm², 4-unit FDPs need 12.6 mm², and 5-unit FDPs should have at least 18.8 mm². The data emphasize that longer spans demand disproportionately larger connectors to ensure reliability, likely due to the increased bending moments and flexural stresses associated with extended FDPs [5].

Research has also highlighted that the connector, being the thinnest cross-section of an FPD, experiences the highest strain and is the most likely point of failure. A slight increase in connector size can significantly enhance the overall strength of an all-ceramic bridge by mitigating stress concentration in this region [13].

Finite element analysis has further underscored the importance of connector shape. It was found that a connector area of approximately 5.3 mm²—automatically determined by certain CAD software—would likely fail under a 500 N load due to von Mises stresses exceeding the material’s yield strength [31]. However, modifying the connector to a more elliptical shape and adding a fillet at the connector–abutment junction effectively reduced peak stresses [32]. It is worth noting that not all studies fully align on this matter.

Some studies suggested that the thickness of the retainer crowns may be more critical than connector dimensions in determining the strength of long-span FDPs. One study indicated that, particularly for anterior FDPs, a smaller connector size of around 9 mm² could be used to improve pontic esthetics without significantly compromising function, provided the retainer crowns maintain sufficient thickness [33].

Additionally, another study demonstrated that connector shape plays a crucial role in structural performance. It was found that a connector with dimensions of 2 mm in height and 3 mm in width, incorporating a 0.6 mm fillet radius at the corners, exhibited greater fracture resistance than a 3 mm × 3 mm connector with a sharp 0.1 mm radius. This finding is particularly relevant in clinical applications, as it suggests that rounding the connector corners (increasing the radius of curvature) can allow for a slightly smaller connector while still maintaining adequate strength in space-constrained situations [34].

This in vitro study has certain limitations that must be considered. The experimental setup used static vertical loading to failure, which does not fully replicate the complex intraoral environment. In the mouth, prostheses are subjected to cyclic loading (chewing forces over time), lateral and angled forces, thermal fluctuations, and the influence of surrounding tissues (periodontal ligament or implant–bone interface compliance). Additionally, only one type of zirconia material and one overall FPD design were tested. Future studies should include fatigue testing (to mimic cyclic chewing stresses) and consider the influence of thermocycling to better approximate oral conditions.

## Conclusions

Based on the findings of this in vitro study, it can be concluded that increasing the connector cross-sectional area and using an oval connector shape both enhance the fracture resistance of long-span monolithic zirconia FPDs. In particular, a 9 mm² connector (3 mm × 3 mm) in a four-unit monolithic zirconia FPD should be used with caution, as it was associated with lower fracture strength and connector-area failures. A 12 mm² connector (3 mm × 4 mm) provided significantly greater strength and shifted the failure origin away from the connector, suggesting it is a safer choice for long-span prostheses. Likewise, an oval connector cross-section is preferable to a circular cross-section because it distributes stress more effectively and improves load-bearing capacity.

## Data Availability

Data is provided within the manuscript.

## Abbreviations

FPD: Fixed Partial Denture
Y-TZP: Yttria-stabilized Tetragonal Zirconia Polycrystal
CAD/CAM: Computer-Aided Design / Computer-Aided Manufacturing
SEM: Scanning Electron Microscopy
STL: Standard Tessellation Language (file format used in 3D modeling)
µm: Micrometer
kN: Kilonewton
N: Newton
